# A GIS-enabled workflow for georeferenced veterinary KAP surveys: a proof-of-concept study in Kazakhstan

**DOI:** 10.3389/fvets.2026.1940797

**Published:** 2026-10-28

**Authors:** Ainur Nurpeisova, Zhandos Abay, Raikhan Nissanova, Anel Bekbergen, Vladislava Suchshikh, Malik Yussupov, Kobeikhan Begassyl, Aspen Abutalip, Markhabat Kassenov, Nurkuisa Rametov

**Affiliations:** 1 Kazakh Scientific Research Veterinary Institute LLP, Almaty, Kazakhstan; 2 Research and Development Department, Tecton Analytics LLP, Astana, Kazakhstan

**Keywords:** ArcGIS Survey123, GIS, Kazakhstan, knowledge, attitude and practice (KAP), veterinary surveillance

## Abstract

One Health surveillance at the animal–human–environment interface requires practical tools that integrate epidemiological, behavioral, and geographic information to support evidence-based decision-making. This study presents a GIS-enabled workflow for integrating georeferenced Knowledge, Attitude, and Practice (KAP) surveys into veterinary surveillance in Kazakhstan. Using ArcGIS Survey123, disease-specific questionnaire modules were developed for anthrax, blackleg, and mastitis and implemented as a proof-of-concept field application. The framework integrates mobile data collection, automated geolocation, standardized KAP indicators, quality-control procedures, livestock-density context, and GIS-enabled spatial visualization within a unified digital workflow. Rather than providing a nationally representative assessment of livestock owners’ knowledge and practices, the study demonstrates the feasibility of combining georeferenced behavioral data with disease-specific veterinary surveillance layers to support spatially informed interpretation and decision-making. The resulting GIS framework illustrates how KAP information can be linked with epidemiological and geographic datasets to support targeted risk communication, farmer education, vaccination planning, and veterinary outreach. As a proof-of-concept, the proposed workflow provides a practical methodological foundation for veterinary surveillance and has potential for future One Health applications through integration with human-health, environmental, and climatic data.

## 1 Introduction

Effective surveillance of animal diseases is a cornerstone of the One Health approach, which recognizes the close interdependence of animal, human, and environmental health. In livestock-producing regions, timely detection and control of infectious and production-related diseases rely not only on laboratory diagnostics, vaccination programs, and veterinary infrastructure but also on the knowledge, attitudes, and practices (KAP) of livestock owners. Farmers’ decisions regarding disease recognition, reporting, vaccination, carcass disposal, biosecurity, and herd management directly influence disease transmission, public health risks, food safety, and the effectiveness of veterinary interventions.

This interconnectedness is particularly important in Central Asia, where extensive livestock production, transboundary animal movements, diverse ecological conditions, and heterogeneous access to veterinary services create complex epidemiological settings. Strengthening disease preparedness therefore requires surveillance systems that integrate epidemiological, behavioral, and spatial information within an integrated veterinary surveillance approach.

In veterinary public health, KAP surveys are particularly important because many animal diseases are controlled at the interface between official veterinary systems and farm-level decision-making (1, 2). Farmers’ awareness of clinical signs, understanding of vaccination, willingness to notify veterinary authorities, and implementation of preventive measures directly influence early detection, outbreak response, and the practical success of disease-control programs. This is especially relevant in extensive livestock-producing regions, where farms and settlements may be geographically dispersed and access to veterinary infrastructure may vary considerably (3–5).

However, conventional KAP surveys remain limited when the geographic context of responses is not considered. Disease awareness, access to veterinary services, livestock management practices, and preventive behaviors are rarely distributed uniformly across rural areas. (6–8). They may differ according to settlement location, livestock density, grazing systems, farm structure, animal movement routes, environmental conditions, and previous experience with disease outbreaks. As a result, questionnaire responses analyzed only as aggregated statistics may fail to identify where knowledge gaps, weak reporting behavior, or insufficient preventive practices are spatially concentrated (9, 10).

Geographic Information Systems (GIS) have evolved beyond mapping tools into decision-support technologies that integrate epidemiological, behavioral, environmental, and geospatial information for evidence-informed disease surveillance and One Health planning.

Recent advances in mobile GIS platforms have further improved the practical implementation of veterinary field surveys. Applications such as ArcGIS Survey123 enable standardized questionnaire design, georeferenced data collection, automated synchronization, skip logic, multimedia documentation, and rapid integration of field records into spatial databases (11–13). These functions are particularly valuable for veterinary fieldwork in large rural territories, where paper-based surveys may be affected by transcription errors, delayed data entry, incomplete geographic attribution, and difficulties in monitoring data quality. Mobile GIS-enabled workflows therefore improve both the operational efficiency of field data collection and the spatial usability of veterinary epidemiological datasets (14–16).

Kazakhstan represents a particularly relevant setting for GIS-enabled veterinary surveillance because of its extensive livestock production systems, large rural territories, transboundary animal movements, and heterogeneous access to veterinary infrastructure (17–21). In such settings, livestock population and density data, particularly for cattle and small ruminants, can serve as important contextual spatial layers for interpreting the veterinary significance of KAP survey coverage and mapped disease-awareness indicators (22). These layers do not replace epidemiological analysis but help identify areas where knowledge gaps or weak preventive practices may be more relevant for veterinary planning.

Anthrax, blackleg, and mastitis were selected because they represent complementary veterinary health challenges relevant to livestock production and disease prevention (23, 24). Anthrax is a zoonotic disease of major One Health significance, whereas blackleg remains an important vaccine-preventable livestock infection (25, 26). Mastitis represents a management-associated disease affecting animal health, productivity, and farm sustainability (27).

Previous KAP studies conducted in Kazakhstan have highlighted the importance of farmer awareness, local management practices, and communication between livestock owners and veterinary services for effective disease prevention and control (28–30). However, although KAP surveys and GIS technologies have each been widely applied in veterinary epidemiology, only a limited number of studies have integrated georeferenced behavioral data, disease-specific surveillance information, and spatial decision-support tools within a unified veterinary surveillance framework.

This gap remains particularly evident in Central Asia, where practical digital surveillance approaches remain limited despite the region’s importance for transboundary animal health and zoonotic disease preparedness (31, 32).

The objective of this study was to develop and demonstrate the feasibility of a GIS-enabled workflow for integrating georeferenced KAP surveys into veterinary surveillance in Kazakhstan. Rather than providing a nationally representative assessment of livestock owners’ knowledge and practices, the study demonstrates the feasibility of combining mobile GIS technologies, disease-specific KAP modules, and spatial epidemiological information within a unified veterinary decision-support workflow. Anthrax, blackleg, and mastitis were used as proof-of-concept case studies. The anthrax module provides particular One Health relevance because of its zoonotic significance, while integration with human-health, environmental, and climatic datasets remains a prospective application of the workflow.

## 2 Materials and methods

### 2.1 Study design and general framework

This study developed and applied a GIS-enabled Knowledge, Attitude, and Practice (KAP) survey framework for veterinary epidemiological mapping in Kazakhstan. The framework integrated structured questionnaire-based data collection with mobile geospatial technologies and GIS-enabled visualization to assess disease awareness, preventive practices, and spatial patterns associated with three livestock diseases: anthrax, blackleg (emphysematous carbuncle), and mastitis.

A cross-sectional field survey design was implemented using ArcGIS Survey123. The workflow comprised questionnaire development, digital form configuration, mobile georeferenced data collection, cloud synchronization, quality control, and GIS-enabled visualization. Linking questionnaire responses with geographic coordinates and administrative attributes enabled the generation of disease-specific spatial layers and identification of areas requiring targeted veterinary communication and educational interventions.

### 2.2 Study area and target population

The survey was conducted in selected livestock-producing regions of Kazakhstan based on their relevance to veterinary surveillance and disease mapping. The target population included livestock owners, smallholder farmers, and individuals involved in animal husbandry and farm management. Surveyed communities were selected to represent areas where anthrax, blackleg, and mastitis are of veterinary, epidemiological, or economic importance. The selected diseases represent distinct epidemiological contexts. Anthrax and blackleg are environmentally associated infectious diseases requiring continuous veterinary monitoring, whereas mastitis is a production-related disease of dairy cattle with substantial economic impact. Inclusion of these disease modules enabled evaluation of the GIS-supported KAP system across both infectious and management-associated animal health challenges. Field data were collected during the spring–summer season of 2025. A total of 300 livestock owners and farm representatives participated in the survey. Participants were recruited using non-probability convenience sampling among eligible individuals who were available during field visits and agreed to participate. Eligibility was based on direct involvement in livestock ownership, animal husbandry, or farm management. The proof-of-concept dataset comprised 33 georeferenced locations across the three disease-specific modules (anthrax, *n* = 6; blackleg, *n* = 19; mastitis, *n* = 8). These locations were used to demonstrate implementation of the GIS-enabled framework and were not intended to constitute a nationally representative spatial sample. The 300 participants represented the overall questionnaire sample, whereas the 33 georeferenced locations represented the spatial units used for GIS visualization and were not equivalent to individual respondents. The primary unit for mapping was the georeferenced survey location.

### 2.3 Questionnaire structure and KAP indicators

The questionnaire followed the conventional KAP structure and included sections on socio-demographic characteristics, livestock ownership, disease-related knowledge, attitudes toward disease prevention and veterinary control, and reported management practices. Variables included province, district, participant information, age, gender, education level, occupation, and livestock species kept on the farm.

Responses were coded prior to spatial analysis to ensure standardized interpretation across disease modules. Socio-demographic and farm-related variables were treated as explanatory factors, whereas disease-specific items were grouped into knowledge, attitude, and practice domains. Figure 1 presents the coding structure using the anthrax module as an example; the same analytical workflow was subsequently adapted for blackleg and mastitis.

**Figure 1:**
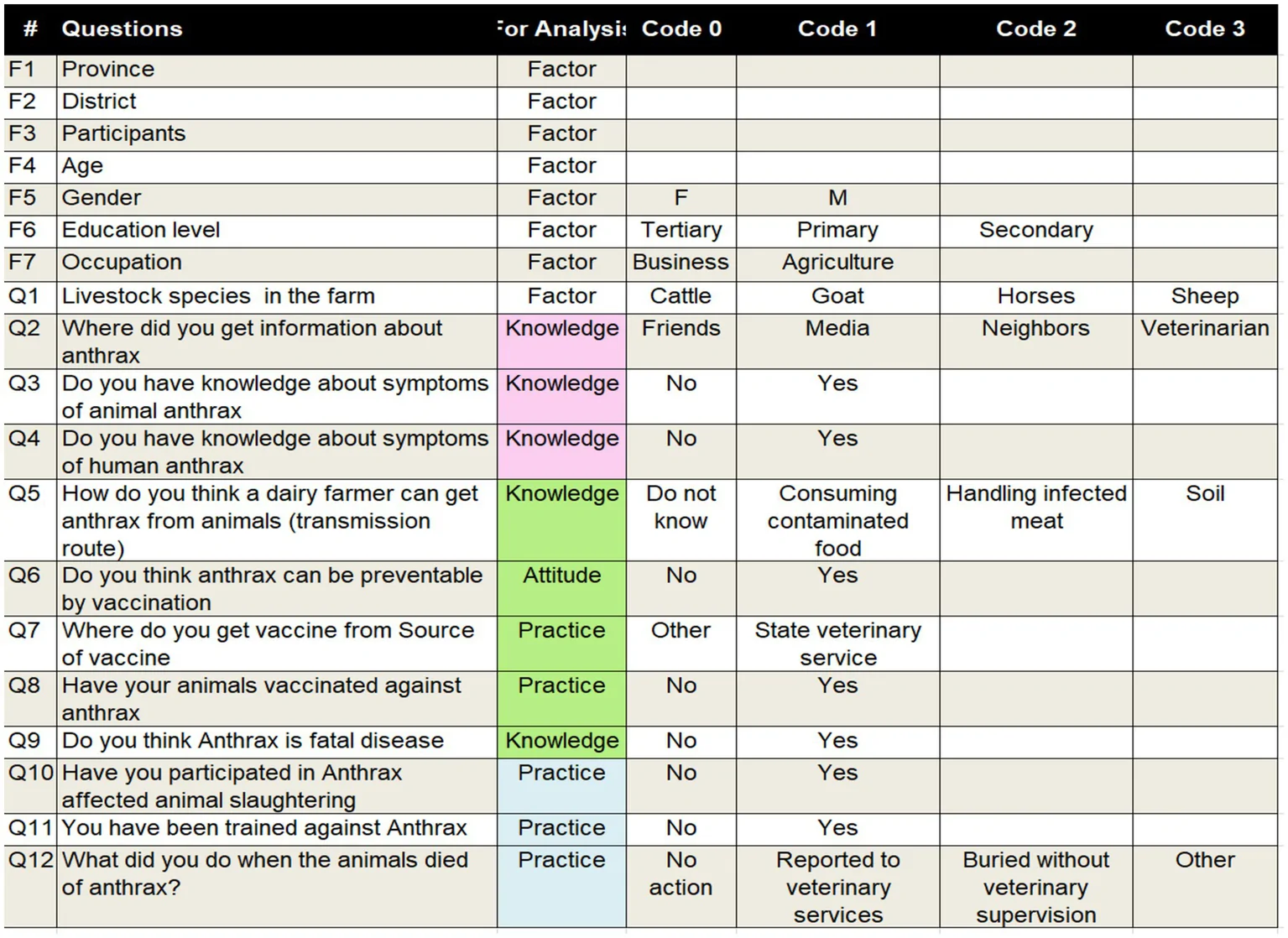
Structure and coding scheme of the GIS-enabled veterinary KAP questionnaire used for disease-specific data collection and spatial epidemiological analysis. The questionnaire was organized into Factor, Knowledge, Attitude, and Practice (KAP) domains. Factor variables included respondent demographics, geographic location, and farm characteristics. Knowledge questions assessed awareness of disease symptoms and transmission pathways, Attitude questions evaluated perceptions of disease prevention and veterinary services, and Practice questions captured preventive behaviors, participation in veterinary interventions, and responses to disease events. Response categories were standardized using predefined coding schemes to facilitate data management, GIS integration, and comparative analysis across disease-specific modules. The Figure reflects the organizational layout of the original field questionnaire; analytical assignment of individual items to Knowledge, Attitude, and Practice domains was based on the construct represented by each question, as described in Section 2.3.

The questionnaire was developed for proof-of-concept implementation and was not subjected to formal psychometric validation or reliability testing; this limitation should be considered when interpreting the KAP indicators. Figure 1 reproduces the organizational structure of the original digital questionnaire used during field implementation. For analytical interpretation, individual items were assigned to KAP domains according to the construct they represented; thus, questions addressing disease transmission or severity were treated as Knowledge items, whereas questions describing actual vaccination-related behavior were interpreted as Practice items. Question Q12 was classified as a categorical Practice indicator with four response options: 0 = No action, 1 = Reported to veterinary services, 2 = Buried without veterinary supervision, and 3 = Other. These categories were retained for descriptive interpretation of reported actions. Reporting to veterinary services was distinguished from inaction and burial without veterinary supervision, while “Other” responses were retained separately. No binary safe/unsafe carcass-disposal score was calculated.

The disease-specific questionnaire items were selected to reflect epidemiologically relevant knowledge, attitudes, and preventive practices commonly assessed in veterinary KAP studies and were informed by previously published KAP frameworks addressing livestock disease awareness, vaccination, reporting behavior, biosecurity, and disease-prevention practices (1, 2, 14–16, 28–30). The indicators were adapted to the epidemiological and management characteristics of anthrax, blackleg, and mastitis and were used for descriptive and spatial interpretation within the proof-of-concept framework.

### 2.4 Digital survey development using ArcGIS Survey123

ArcGIS Survey123 was used as a form-centric data collection application for implementing the proof-of-concept framework and supporting standardized mobile and georeferenced data collection. The digital form included closed-ended questions, coded response options, mandatory fields, skip logic, geolocation capture, date and time recording, and optional multimedia attachments. The platform was used as a technical environment for implementing the proposed workflow rather than as the object of evaluation itself. Survey123 was selected because it supports offline data collection, automated synchronization with ArcGIS Online, and direct integration of survey responses with geographic coordinates. These features are expected to reduce transcription errors, support data completeness, and facilitate more rapid transfer of field data into the central GIS database.

### 2.5 Field data collection and quality control

Field data were collected using preconfigured Survey123 forms on mobile devices. Prior to deployment, field personnel were trained on questionnaire administration, response coding, geolocation settings, confidentiality requirements, and standardized interviewing procedures. Each completed questionnaire was automatically linked to geographic coordinates, administrative attributes, and the corresponding disease module. Survey records were synchronized with the ArcGIS cloud environment when internet connectivity was available. Quality-control procedures included verification of completeness, duplicate detection, coordinate validation, and consistency checks for administrative attribution and survey responses.

### 2.6 Data processing and KAP classification

Survey responses were exported from ArcGIS Survey123 and organized into disease-specific datasets for anthrax, blackleg, and mastitis. Responses were coded according to the predefined answer categories included in the digital questionnaire. Socio-demographic variables were treated as explanatory or stratification factors, while knowledge, attitude, and practice items were grouped according to their respective KAP domains.

For spatial visualization, selected KAP indicators were summarized at the level of survey points and, where appropriate, aggregated by administrative units. Disease-specific layers were prepared to display the geographic distribution of survey responses and mapped indicators. The classification of responses was used to identify areas with lower disease awareness, insufficient preventive practices, or higher need for targeted veterinary communication. Individual KAP indicators were coded and visualized according to their predefined response categories. Where several indicators were considered jointly, they were used for descriptive spatial interpretation rather than for calculation of a validated composite KAP score. Regional surveillance-priority categories (Very High, High, Moderate, Low, and Very Low) presented in Table 1 were assigned using expert judgment integrating livestock population density, spatial occurrence of disease-associated locations, epidemiological importance of the disease, and expected priority for veterinary surveillance and preventive interventions. These categories were used for qualitative decision support and should not be interpreted as quantitative risk estimates. Survey locations were used as the primary spatial unit, while administrative-level aggregation was used only for descriptive visualization where appropriate. KAP information was considered as complementary contextual information and was not used as a quantitative weighting variable in the assignment of regional surveillance-priority categories.

**Table 1:** Integrated spatial interpretation of livestock density and disease-specific veterinary surveillance priorities in Kazakhstan.

Region	Livestock density significance	Anthrax surveillance relevance	Blackleg surveillance relevance	Mastitis surveillance relevance
Turkistan	Very high	Very high	Moderate	Low
Almaty	Very high	High	High	Very high
Zhambyl	High	Very high	Moderate	Moderate
West Kazakhstan	High	Moderate	High	Low
East Kazakhstan	High	Moderate	Moderate	High
Pavlodar	High	Moderate	High	Moderate
Abai	High	Moderate	High	Moderate
Karaganda	Moderate	Low	High	High
Kostanay	Low	Low	Moderate	High
Akmola	Moderate	Low	Moderate	Moderate
North Kazakhstan	Moderate	Low	Moderate	Moderate
Aktobe	Moderate	Moderate	Moderate	Low
Kyzylorda	Low	Low	Low	Low
Atyrau	Low	Low	Low	Low
Mangystau	Very low	Low	Low	Low
Ulytau	Very low	Low	Low	Low
Zhetisu	High	Moderate	High	Moderate

The qualitative categories (Very high, High, Moderate, Low, and Very low) were assigned by expert review of regional livestock density, the spatial occurrence of disease-associated locations, and the epidemiological relevance of each disease. No numerical weighting or formal risk-scoring algorithm was applied. KAP information was considered as complementary contextual information and did not independently determine or quantitatively weight the assigned categories. The classification is intended for qualitative decision support and should not be interpreted as a quantitative risk model.

### 2.7 GIS mapping and spatial visualization

Georeferenced survey data were integrated into ArcGIS for spatial visualization and epidemiological mapping. The anthrax contextual layer comprised 33 reported outbreak records from 2015–2024, whereas the anthrax KAP component comprised six georeferenced survey locations. These datasets were maintained as separate layers and were linked spatially within the GIS environment rather than merged at the individual-record level. Survey points were displayed as thematic layers according to disease module and selected KAP indicators. Administrative boundaries, settlement locations, and livestock-density layers were used as contextual geographic references. The GIS workflow included spatial data cleaning, layer generation, symbolization, map composition, and export of publication-ready cartographic outputs. ArcGIS Online and ArcGIS Experience Builder were used for interactive visualization and preliminary inspection of field data, whereas final maps were prepared for manuscript presentation and comparative interpretation of anthrax, blackleg, and mastitis surveillance layers.

### 2.8 Ethical considerations

Participation in the survey was voluntary. Before questionnaire administration, participants were informed about the purpose of the study, the type of information collected, confidentiality, and the intended use of anonymized data for epidemiological mapping and veterinary public health analysis, and participated only after voluntarily agreeing to take part. Personal identifiers were not used in the spatial analysis or in the presentation of results. Geographic data were processed and visualized in a way that supported epidemiological interpretation while maintaining participant confidentiality. The study protocol, including questionnaire-based data collection from livestock owners and farm representatives, was reviewed and approved by the Institutional Bioethics Committee of the Kazakh Scientific Research Veterinary Institute (KazSRVI; Protocol No. 01–24.01.2025, approved 24 January 2025).

## 3 Results

### 3.1 Development of the GIS-enabled KAP survey workflow

A GIS-enabled workflow for veterinary KAP surveillance was successfully implemented using ArcGIS Survey123 and the ArcGIS spatial analysis environment (Figure 2). The workflow integrated digital questionnaire deployment, mobile georeferenced data collection, automated cloud synchronization, quality-control verification, and GIS-enabled visualization within a unified digital platform.

**Figure 2:**
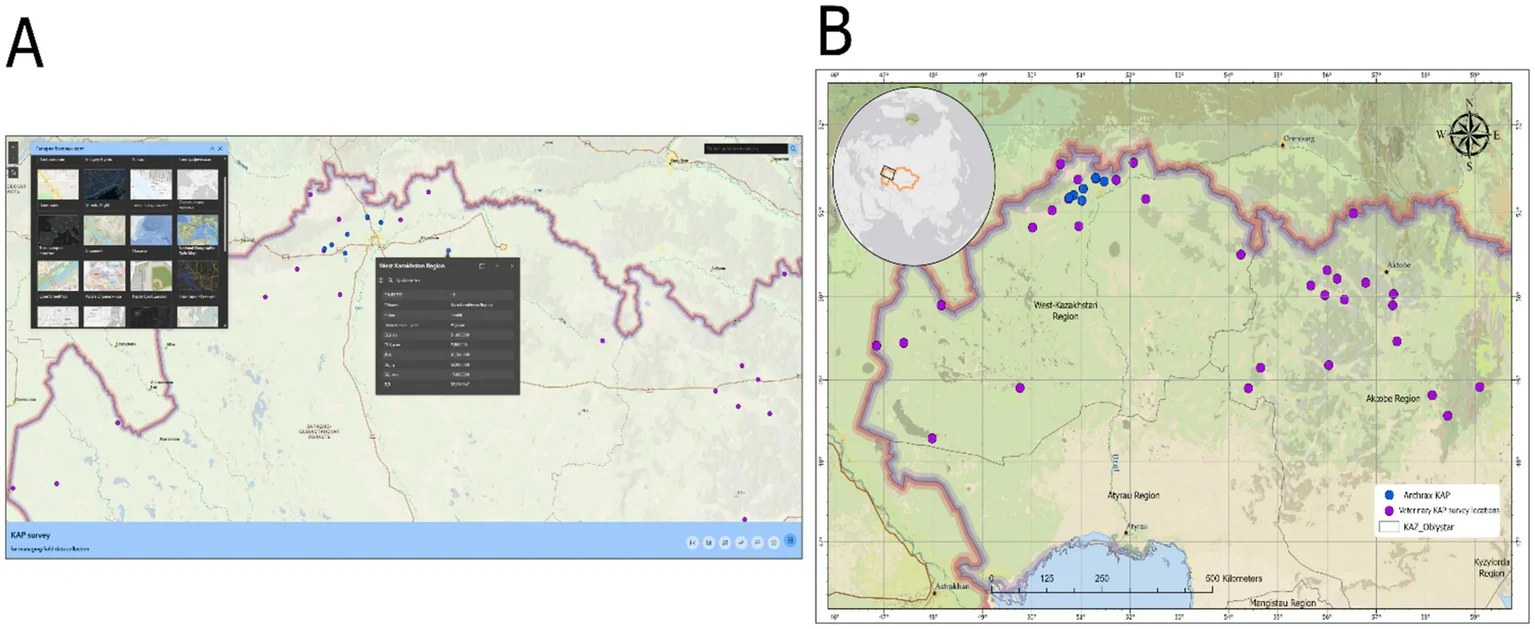
GIS workflow and real-time visualization of georeferenced veterinary KAP survey records in ArcGIS Survey123. **(A)** Real-time synchronization of georeferenced questionnaire records from ArcGIS Survey123 to the ArcGIS environment, enabling real-time visualization, quality-control verification, and monitoring of field data collection. **(B)** Spatial distribution of georeferenced KAP survey records displayed within the GIS environment, illustrating survey coverage and supporting verification of geographic completeness during field implementation.

The system enabled direct collection of questionnaire responses together with geographic coordinates, administrative attributes, temporal metadata, and disease-specific KAP indicators. All records were automatically associated with spatial locations at the time of collection, thereby avoiding a separate post-survey georeferencing step. Real-time synchronization with the ArcGIS environment enabled real-time visualization of incoming survey records, verification of spatial coverage, and identification of incomplete or inconsistent entries. The resulting workflow produced standardized geospatial datasets suitable for disease-specific mapping, descriptive interpretation of KAP indicators, and potential use in veterinary decision-support applications.

### 3.2 Structure of the KAP questionnaire and disease-specific adaptation

The developed questionnaire provided a unified analytical workflow for assessing livestock owners’ knowledge, attitudes, and practices across multiple veterinary disease modules. The survey structure comprised four interconnected components: socio-demographic and farm-related characteristics, knowledge indicators, attitude indicators, and practice indicators (Supplementary Figure 1).

Application of the framework to anthrax, blackleg, and mastitis demonstrated its flexibility across diseases with distinct epidemiological characteristics and surveillance objectives. Although the overall analytical structure remained consistent, disease-specific modules were adapted to reflect differences in transmission pathways, prevention strategies, farm-management practices, and veterinary response requirements.

Digital implementation in ArcGIS Survey123 enabled collection of georeferenced respondent information together with disease-specific KAP indicators. The mobile interface incorporated demographic variables, geographic coordinates, temporal metadata, and disease-related questions within a standardized digital workflow (Supplementary Figures 2, 3).

The resulting digital workflow produced standardized disease-specific datasets that can support GIS-enabled spatial analysis and comparative assessment of KAP indicators across the three disease modules.

### 3.3 Geographic coverage, spatial data integration, and descriptive KAP findings

Field implementation of the GIS-enabled KAP system resulted in a georeferenced database linking questionnaire-derived behavioral information with spatial coordinates and disease-specific contextual data. For the proof-of-concept analysis, the georeferenced KAP survey locations and the disease-associated epidemiological records were treated as distinct but complementary data components. The KAP component retained the geographic context of disease-awareness and preventive-practice information, whereas the disease-specific layers provided the epidemiological context for spatial interpretation. Table 2 summarizes the geographic coverage, KAP information represented in the GIS workflow, and the corresponding contextual disease layers used for the three modules.

**Table 2:** Structure of disease-specific data components used in the GIS-enabled KAP proof-of-concept analysis.

Disease module	Regions represented	Georeferenced KAP survey locations, n	KAP information represented in the GIS workflow	Contextual disease layer used for interpretation
Anthrax	1	6	Disease awareness, vaccination awareness, reporting behavior, and preventive practices	33 reported anthrax outbreak records, 2015–2024
Blackleg	17	19	Disease awareness, vaccination awareness, and preventive behavior	19 *Clostridium chauvoei*-associated location records
Mastitis	4	8	Disease awareness, herd-management indicators, and preventive practices	8 farm-level mastitis-associated records
Total	-	33	-	-

The values in the third column refer to georeferenced survey locations rather than individual respondents. The KAP and disease-associated layers represent distinct data components integrated for descriptive spatial interpretation; the table does not present population-level KAP prevalence estimates.

The three disease-specific survey modules included 100 respondents for anthrax, 110 for blackleg, and 90 for mastitis (Table 3). In the anthrax module, 92.0% (92/100) of respondents reported knowledge of clinical signs, 75.0% (75/100) reported animal vaccination, and 67.0% (67/100) reported previous anthrax-related training. In the blackleg module, reported knowledge of clinical signs and preventive vaccination were 61.8% (68/110) and 49.1% (54/110), respectively, while the mastitis module showed 97.8% (88/90) for reported knowledge of clinical signs and 87.8% (79/90) for preventive herd-management practices. Unlike disease-location records alone, these KAP findings provide additional information on respondents’ reported knowledge and preventive behaviors, which can be interpreted alongside georeferenced surveillance data to identify potential priorities for veterinary education and communication without implying population-level representativeness.

**Table 3:** Selected descriptive KAP findings from disease-specific veterinary surveys in Kazakhstan.

Disease module	KAP indicator	Positive responses, n/N	Percentage (%)
Anthrax	Self-reported knowledge of clinical signs	92/100	92.0
Anthrax	Reported vaccination of animals against anthrax	75/100	75.0
Anthrax	Previous anthrax-related training	67/100	67.0
Blackleg	Self-reported knowledge of clinical signs	68/110	61.8
Blackleg	Reported preventive vaccination	54/110	49.1
Mastitis	Self-reported knowledge of clinical signs	88/90	97.8
Mastitis	Reported preventive herd-management practices	79/90	87.8

Values represent descriptive proportions based on valid responses to selected questionnaire items. The survey used non-probability sampling; therefore, the findings should not be interpreted as population-level prevalence estimates.

Integration of geolocation data transformed questionnaire responses into spatially explicit records and enabled their organization into separate analytical layers for anthrax, blackleg, and mastitis. This structure supported disease-specific interpretation and comparative visualization across the three modules. Quality-control procedures included verification of survey locations, administrative attribution, geographic completeness, and consistency of spatial attributes. Records with missing coordinates or inconsistent spatial information were reviewed and corrected before map generation. The resulting geospatial database provided the basis for disease-specific mapping and descriptive spatial interpretation of KAP indicators.

### 3.4 Spatial visualization of anthrax-related KAP indicators

The anthrax disease module produced a disease-specific GIS layer integrating georeferenced outbreak locations with KAP survey data (Figure 3). Reported anthrax outbreak locations distributed across multiple regions of Kazakhstan and served as epidemiological context for interpretation of disease-awareness and prevention-related indicators.

**Figure 3:**
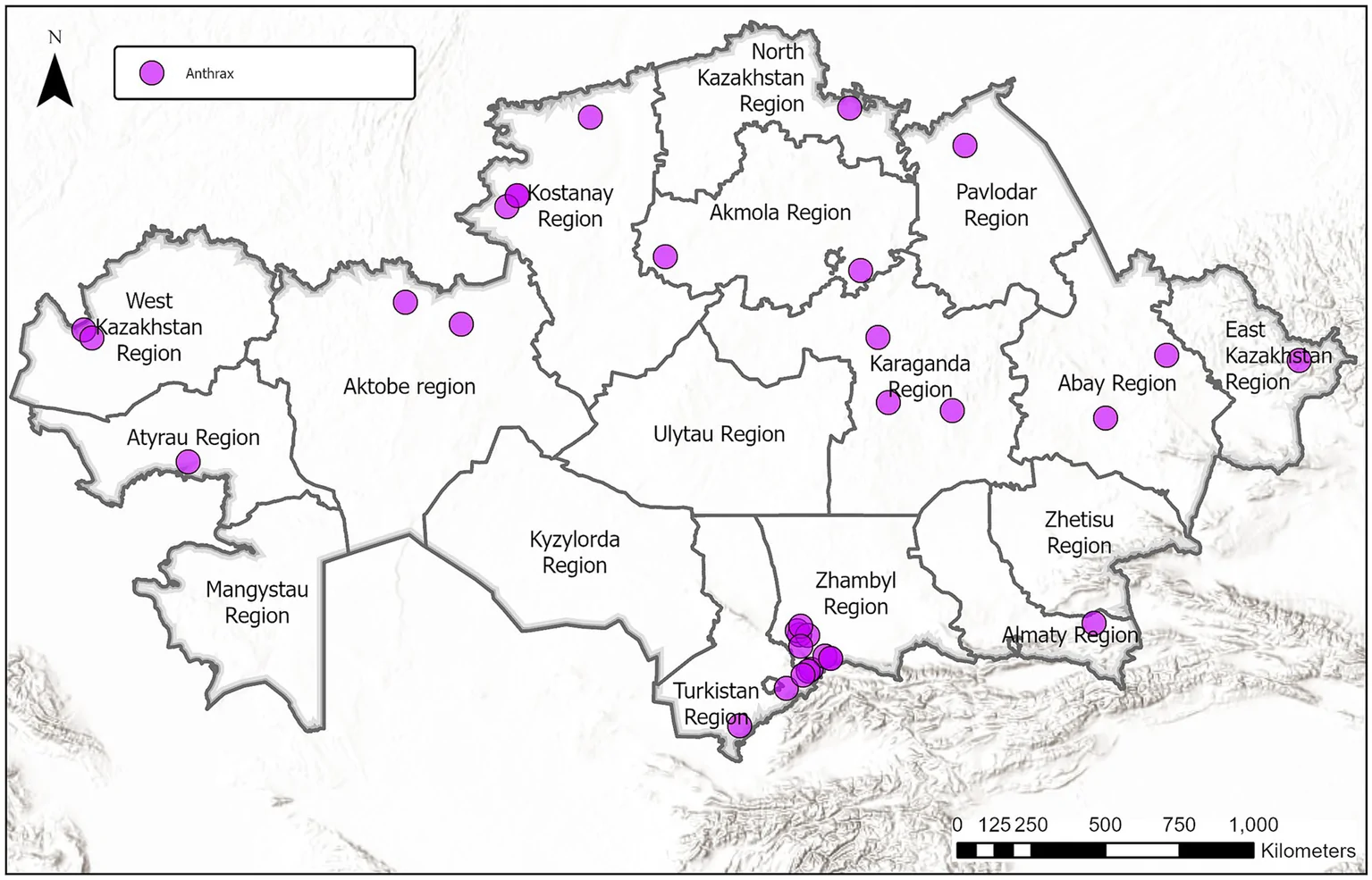
Spatial distribution of reported anthrax outbreak locations in Kazakhstan used as the epidemiological context for GIS-enabled interpretation of veterinary KAP survey data. Purple circles indicate reported anthrax outbreak locations used as reference points for interpreting disease-awareness indicators, vaccination awareness, reporting behavior, and preventive practices within the GIS-enabled KAP framework. The map illustrates the geographic context of the proof-of-concept application and does not represent the complete national distribution or intensity of anthrax outbreaks.

Spatial analysis revealed a heterogeneous geographic distribution of anthrax-associated locations, with the highest concentration observed in southern Kazakhstan, particularly within Turkistan and Zhambyl Regions. Additional outbreak locations were identified in western, northern, central, and eastern parts of the country, indicating the broad geographic relevance of anthrax surveillance and awareness activities. Integration of outbreak-location data with georeferenced KAP records provided epidemiological context for evaluating disease-related knowledge and preventive practices among livestock owners. Areas characterized by a higher density of anthrax-associated locations may represent priority areas for strengthening awareness of disease recognition, vaccination, carcass disposal, and reporting procedures. The resulting GIS layer illustrates how epidemiological information can be integrated with behavioral survey data to support geographically targeted veterinary surveillance, risk communication, and disease-control planning.

### 3.5 Spatial visualization of blackleg-related awareness and prevention practices

The blackleg disease module produced a GIS layer integrating reported *Clostridium chauvoei*-associated locations with georeferenced KAP survey records (Supplementary Figure 4). The resulting spatial dataset supported assessment of disease-related knowledge, preventive behavior, and vaccination practices across surveyed regions.

GIS analysis illustrated the broad geographic distribution of reported blackleg outbreak locations across Kazakhstan. Higher concentrations of reported locations were observed in northern and northeastern regions, whereas additional locations occurred in central, southeastern, and western parts of the country, providing epidemiological context for interpreting georeferenced KAP survey responses.

Integration of epidemiological and georeferenced KAP information demonstrated the feasibility of combining behavioral and spatial datasets within a unified GIS-enabled surveillance system. Areas containing greater numbers of reported outbreak locations may represent priority settings for future vaccination awareness campaigns, farmer education, and veterinary outreach aimed at strengthening disease prevention and early reporting.

Overall, the resulting GIS layer illustrates how epidemiological information can be integrated with georeferenced behavioral data to support evidence-informed veterinary surveillance, targeted risk communication, vaccination planning, and veterinary decision-making.

### 3.6 Spatial visualization of mastitis-related management indicators

The mastitis module produced a disease-specific GIS layer integrating georeferenced mastitis-associated locations with KAP survey information related to herd management and disease prevention (Supplementary Figure 5). GIS mapping illustrated the geographic distribution of mastitis-associated locations across several livestock-producing regions of Kazakhstan, including Kostanay, Karaganda, East Kazakhstan, and Turkistan Regions. These locations provide epidemiological context for interpreting georeferenced KAP survey responses related to herd management, disease awareness, and preventive practices.

Unlike anthrax and blackleg, mastitis is primarily influenced by herd-management practices, milking hygiene, early recognition of clinical signs, and timely veterinary intervention. Accordingly, spatial interpretation of mastitis-related KAP indicators provides a useful system for identifying areas where farmer education, advisory services, and herd-health management strategies may require strengthening.

Integration of disease-location data with GIS-enabled survey information illustrates how production-related animal health problems can be incorporated into spatial veterinary surveillance systems. The resulting GIS layer supports geographically targeted herd-health interventions and highlights the potential of combining epidemiological, behavioral, and spatial information within a unified veterinary decision-support system.

### 3.7 Integrated GIS interpretation of disease-specific veterinary surveillance layers

Integrated analysis of anthrax, blackleg, and mastitis surveillance layers facilitated comparative interpretation of disease-specific KAP indicators within a unified GIS environment. Simultaneous visualization of infectious and management-associated animal health challenges provided an integrated spatial perspective for evaluating disease awareness, preventive behavior, and veterinary intervention priority areas across Kazakhstan.

Figure 4 illustrates the spatial distribution of cattle and small-ruminant population densities across Kazakhstan, providing valuable spatial context for interpreting georeferenced veterinary KAP survey records. Livestock populations were heterogeneously distributed among regions, with higher cattle densities observed in Almaty, Turkistan, West Kazakhstan, and Zhambyl Regions, whereas higher small-ruminant densities occurred predominantly in southern Kazakhstan, particularly in Turkistan, Almaty, and Zhambyl Regions.

**Figure 4:**
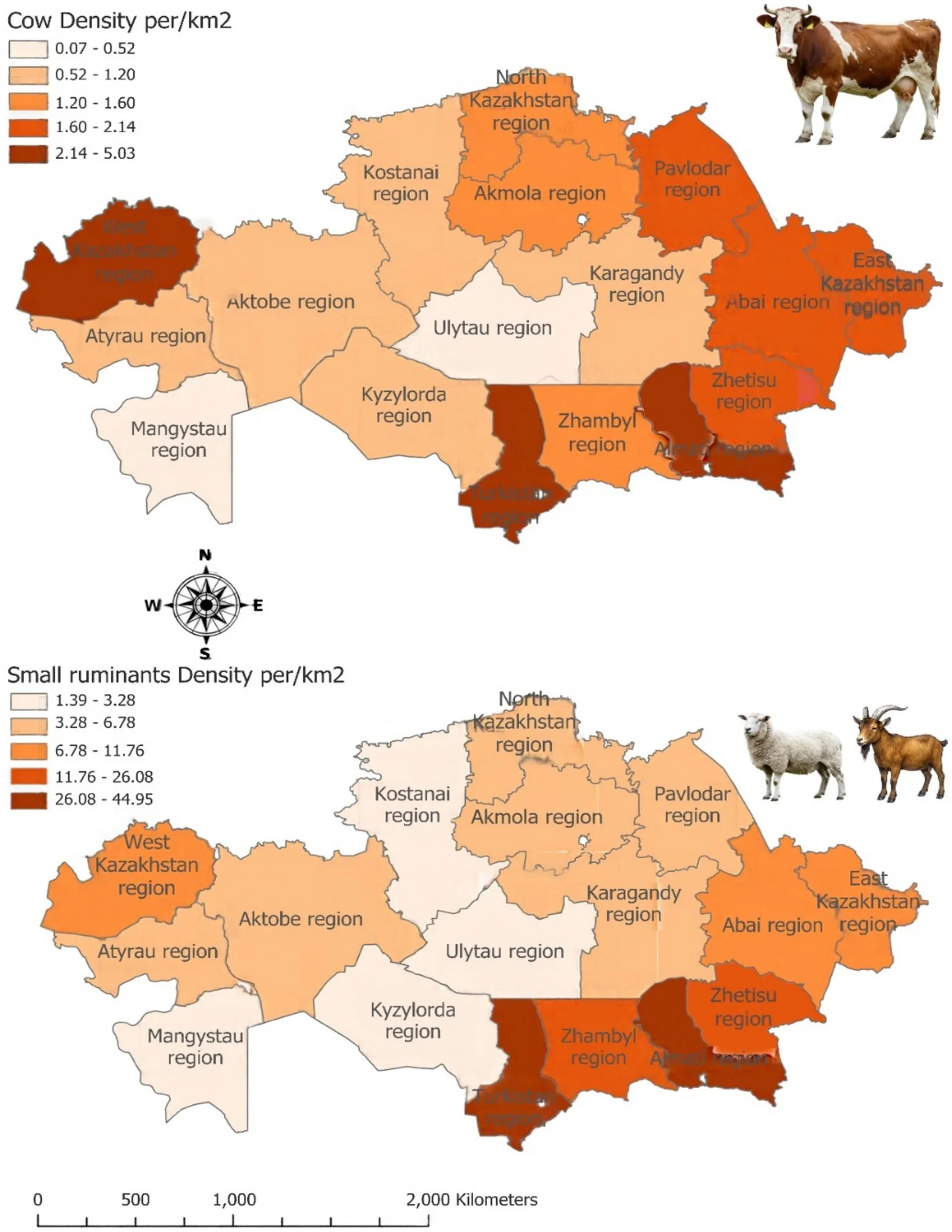
Spatial distribution of cattle and small-ruminant population densities in Kazakhstan used as contextual livestock layers for GIS-enabled interpretation of veterinary KAP indicators. Spatial distribution of small-ruminant population density. Livestock-density layers were incorporated as contextual spatial variables to support interpretation of georeferenced KAP survey records by providing information on the distribution of livestock populations relevant to disease awareness, preventive practices, veterinary communication, and surveillance planning. These layers were used for contextual interpretation within the proof-of-concept framework and were not included as predictive variables in spatial risk modeling.

Comparative spatial analysis revealed distinct geographic patterns among the three disease modules. Anthrax-associated locations formed pronounced clusters in southern Kazakhstan, reflecting areas of historical and contemporary epidemiological relevance. Blackleg-associated locations were more broadly distributed across livestock-producing regions, highlighting the importance of vaccination-based prevention. In contrast, mastitis-associated locations were concentrated in regions characterized by intensive cattle production, highlighting the influence of herd-management practices and dairy production systems on disease occurrence.

Integration of disease-specific surveillance layers with livestock-density information enabled identification of geographic areas where disease occurrence, management practices, and awareness-related factors may converge. Such spatially explicit information can support prioritization of veterinary interventions, educational programs, vaccination campaigns, and risk-communication activities.

Collectively, integration of multiple disease modules within a unified GIS environment highlights the value of combining epidemiological, behavioral, and geographic information for veterinary decision support. The resulting framework provides an approach with potential for future scaling for geographically targeted livestock health management and evidence-based disease-control planning in Kazakhstan.

### 3.8 Practical utility of GIS-enabled veterinary surveillance

Integration of georeferenced KAP survey data with GIS-enabled spatial analysis enabled the generation of disease-specific surveillance layers for anthrax, blackleg, and mastitis. The resulting maps supported interpretation of disease-awareness patterns, preventive practices, and management-related factors within their geographic context.

Spatial integration illustrated how areas with potential needs for strengthened veterinary communication, vaccination awareness, preventive measures, or herd-health management could be identified. The workflow was not used to evaluate specific veterinary interventions or management decisions in the present study. Rather, the resulting geospatial information demonstrates its potential to support future farmer education, risk communication, veterinary outreach, and surveillance planning. For veterinary services, this integration enables geographically explicit identification of areas where gaps in disease awareness, vaccination-related knowledge, preventive practices, or herd-health management may require attention. Such information can help prioritize farmer education, risk communication, veterinary outreach, and allocation of surveillance activities to locations where they are most needed. The resulting geospatial outputs illustrate the potential of GIS-enabled KAP mapping to inform geographically targeted disease-control activities, veterinary resource prioritization, and livestock-health management.

## 4 Discussion

This study demonstrates the feasibility of integrating mobile GIS technologies with KAP-based veterinary surveillance in Kazakhstan. The demonstrated implementation was veterinary in scope; no direct human-health, environmental, or climatic datasets were integrated, and no formal cross-sectoral decision-making was undertaken. The One Health relevance is most evident in the anthrax module because of the zoonotic nature of the disease and the potential for future linkage of veterinary KAP data with public-health and environmental information. Rather than providing a nationally representative assessment of livestock owners’ knowledge and practices, the proposed approach serves as a proof-of-concept for combining georeferenced behavioral data with disease-specific epidemiological information in a unified decision-support environment. By combining animal health, behavioral, and spatial information, this approach supports spatially informed surveillance, risk communication, and veterinary planning, while providing a methodological foundation for larger and more representative studies and for future One Health applications in Kazakhstan and across Central Asia.

One of the principal findings of this study is that ArcGIS Survey123 provided a practical platform for standardized KAP data collection while preserving spatial specificity. The integration of geolocation, coded response fields, skip logic, cloud synchronization, and GIS visualization provided the technical basis for standardized georeferenced data collection. These features may help reduce limitations commonly associated with paper-based surveys, including transcription errors, delayed data entry, and incomplete geographic attribution; however, these operational benefits were not quantitatively evaluated in the present study. The methodological contribution of the study therefore lies not in the Survey123 software itself, but in the integration of disease-specific KAP questionnaires, geolocation, quality-control procedures, and contextual epidemiological layers within a reproducible veterinary surveillance workflow.

The three disease modules represented distinct veterinary and epidemiological contexts. Anthrax emphasized the importance of disease awareness, reporting behavior, carcass-disposal practices, and vaccination awareness in historically affected areas. Blackleg emphasized the continuing importance of preventive vaccination and farmer education, whereas mastitis represented the critical role of herd management, milking hygiene, and early recognition of clinical signs in dairy-production systems. Collectively, these modules demonstrated the adaptability of the GIS-enabled approach across both infectious and management-associated animal health challenges.

These observations are consistent with previous studies demonstrating that the integration of behavioral and spatial information can strengthen animal disease surveillance and veterinary decision-making. Compared with conventional KAP surveys, which generally summarize responses at the population level, the proposed GIS-enabled system retains the geographic context of each survey record, allowing behavioral information to be interpreted alongside epidemiological and livestock-distribution data. Although evaluated here using three veterinary disease modules, the approach has considerable potential for supporting risk communication, vaccination planning, participatory surveillance, and multisectoral One Health collaboration in livestock-producing regions of Central Asia.

Several limitations should be acknowledged. First, the study was designed on demonstrating the integration of GIS and KAP methodologies rather than on advanced spatial risk modeling. Second, the anthrax, blackleg, and mastitis modules were included as proof-of-concept case studies and were not intended to estimate nationally representative levels of awareness or management practices. Third, KAP responses were based on self-reported information and may therefore be influenced by recall bias or social desirability bias. Finally, livestock-density layers were used as contextual variables and were not incorporated into formal predictive spatial models. The number of georeferenced survey records was limited because the primary objective of the study was to evaluate the feasibility of the GIS-enabled KAP system rather than to conduct a nationally representative epidemiological survey. Future studies should further evaluate the system using larger and geographically representative datasets and explore the integration of additional environmental, climatic, livestock-movement, and public-health data to further strengthen One Health surveillance.

Notwithstanding these limitations, the study offers a reproducible system for integrating mobile GIS technologies into veterinary KAP surveillance. The successful application of the framework to anthrax, blackleg, and mastitis demonstrates its flexibility across diverse animal-health challenges. The proposed approach may contribute to the development of One Health-oriented surveillance systems by integrating behavioral, epidemiological, and geographic information within a unified decision-support environment.

## 5 Conclusion

This study developed a GIS-enabled workflow combining georeferenced questionnaire data, disease-specific KAP indicators, and spatial analysis for veterinary surveillance in Kazakhstan. Application to anthrax, blackleg, and mastitis demonstrated the feasibility of integrating behavioral, epidemiological, and geographic information within a unified GIS environment. The workflow provides a basis for geographically targeted veterinary surveillance, risk communication, and disease-control planning. Its extension to One Health applications will require future integration with human-health, environmental, climatic, and other cross-sectoral data.

## Data Availability

The original contributions presented in the study are included in the article/Supplementary material, further inquiries can be directed to the corresponding authors.
